# Evaluating a novel sign’s impact on whether park visitors enter a dangerous river

**DOI:** 10.1186/s40621-019-0222-y

**Published:** 2019-11-28

**Authors:** Deborah C. Girasek

**Affiliations:** Department of Preventive Medicine and Biostatistics, Uniformed Services University of the Health Sciences, 4301 Jones Bridge Road, Bethesda, MD 20814 USA

**Keywords:** Risk communication, Drowning, Hikers, National parks, Rivers

## Abstract

**Background:**

Between 1972 and 2015, 56 visitors to the two national parks that border the Potomac River Gorge experienced fatal drowning. In 2016, the George Washington Memorial Parkway (GWMP), and the Chesapeake and Ohio National Historical Park (CHOH) partnered with a researcher to see if enhancement of their risk communication strategies could reduce behaviors that contribute to these deaths.

**Methods:**

An experimental sign, which informed visitors that water entry was illegal and could result in a fine exceeding $200 was developed, and displayed on alternating weekend days from July 30 to September 11, 2016. Those signs were displayed at each park’s entrance, on restroom doors, at trailheads, and at both shorelines of the Potomac. At other times the experimental signs were covered, but a standard safety sign was always present. Cameras were used to record water entries.

**Results:**

Cameras captured 1441 images. Approximately 2% of the images in CHOH and 1% of the images in GWMP showed a visitor in the water*.* Our multivariate analysis revealed that air temperature, beach count, and sign condition were significantly associated with water entry. When our experimental sign was displayed, the odds of an image showing someone in the water was reduced by 63%.

**Conclusions:**

A sign alerting park visitors to the fact that water entry is illegal, and could potentially result in a considerable fine, was associated with significantly reduced risk-taking. While intuitive, this finding is a reminder to consider whether warnings that focus on non-health consequences might be more salient to at-risk populations.

## Background

In recreational settings, signs are often used to communicate rules and warnings to members of the public. This approach may be particularly appealing along long shorelines that cannot be patrolled with regularity. Skeptics might be surprised to learn that numerous studies have shown that signs can influence behavior (Hockett and Hall 2007; Johnson and Swearingen 1992; Lawson and Reigner 2009; Meis and Kashima 2017; Winter 2006; Marschall et al. 2017), especially when attention is paid to evidence-based design (Andrew 2011; Mazis and Morris 1999; Weiler et al. 2015; Williams and Noyes 2007). The effectiveness of varied appeals were addressed in several of these studies but most often they focused on protecting natural resources. In the current research, our interest was in changing visitor behavior to reduce their risk of drowning in the Potomac River.

The 22.5 km Potomac River Gorge is bordered by two national parks: George Washington Memorial Parkway (GWMP) in Virginia and the Chesapeake and Ohio National Historical Park (CHOH) in Maryland. Its waters are deep, with powerful, fast-moving currents and submerged hazards. Per Code of U.S. Federal Regulations [36 CFR 7.96(e)] it is illegal to enter the Potomac River to swim or wade from Federal land, although visitors are permitted to participate in other water activities such as kayaking and fishing. Violators of this statute can receive a fine of more than $200.

Fifty-six people experienced fatal drowning in the Potomac River Gorge between 1972 and 2015 (United States Park Police, Potomac River Fatal Drowning 1975-2015. National Park Service internal report. Unpublished (n.d.)). In an analysis of one year’s worth of water incidents, most involved males aged 15–24, who were swimming or wading from shore, on weekends in the late spring and summer. (Hysi et al. 2017) The parks in question provide water safety information on their websites, through signs posted on-site, by disseminating information through mass and social media, via staff trained to educate members of the public, and in partnership with community groups to maximize the likelihood that visitors will enjoy a safe visit.

After a process that is described in our Methods section, a decision was made to evaluate signage that informed park visitors of the potential legal and financial risks posed by river entry. Several studies conducted in outdoor settings support the use of “sanction” signage (Johnson and Swearingen 1992; Martin 1992). Such channels inform the public that they risk official, negative consequences if they engage in the proscribed behavior.

Our approach was also influenced by the fact that it can be difficult to change people’s risk perceptions, especially with a brief intervention like a sign. In the traffic safety arena, it has been demonstrated that injury risk behaviors can be reduced when audiences are exposed to messages that focus on financial and social consequences (Nichols and Ledingham 2008; Solomon et al. 2004). General Deterrence Theory posits that fear of legal punishment can be effective when people perceive the cost of engaging in an illegal activity to be high (Guttman 2014). We had the option of evaluating a sanction warning because water entry (from land) was already illegal in the national parks where our study was conducted. That provided an opportunity to remind potential risk-takers of a negative consequence they could experience if they entered the river. A recent Canadian study of men who took risks while boating supported the motivational effects of a similar approach (Canadian Safe Boating Council 2014). Safety proponents are in effect competing with the inherent refreshment that attracts recreationalists to water in the heat of summer. Males are at highest risk of drowning generally, within U.S. national parks and at our study sites. Fear appeals have been shown to be less effective with males than females (Tannenbaum et al. 2015).

Since research demonstrates that visitors give little time and attention to signs (Hall et al. 2010), we crafted a message that was very short and clear. This strategy is somewhat novel in national parks, which often list many rules and regulations on a single sign. That results in a busy presentation of messages which vary in importance. “Visual clutter” has been shown to reduce the noticeability of warnings (Wogalter et al. 2005). Our background sign incorporated the signal word “Danger,” and the color red because they have been shown to communicate serious hazards to the public (Wogalter et al. 2005). It was also posted where visitors would decide whether to enter the water.

The primary purpose of our investigation was to answer the question: Does the addition of signage that describes the potential legal & financial consequences that violators face for entering the Potomac River, have any impact on visitor water entries? A secondary goal of our research was to explore whether environmental conditions at the study sites were associated with increased odds of river entry. (In a previous study, carried out at Yosemite National Park, visitors’ river entries were associated with air temperature and water level.) (Girasek et al. 2016a)

## Study Design & Methods

**Fig. 1:**
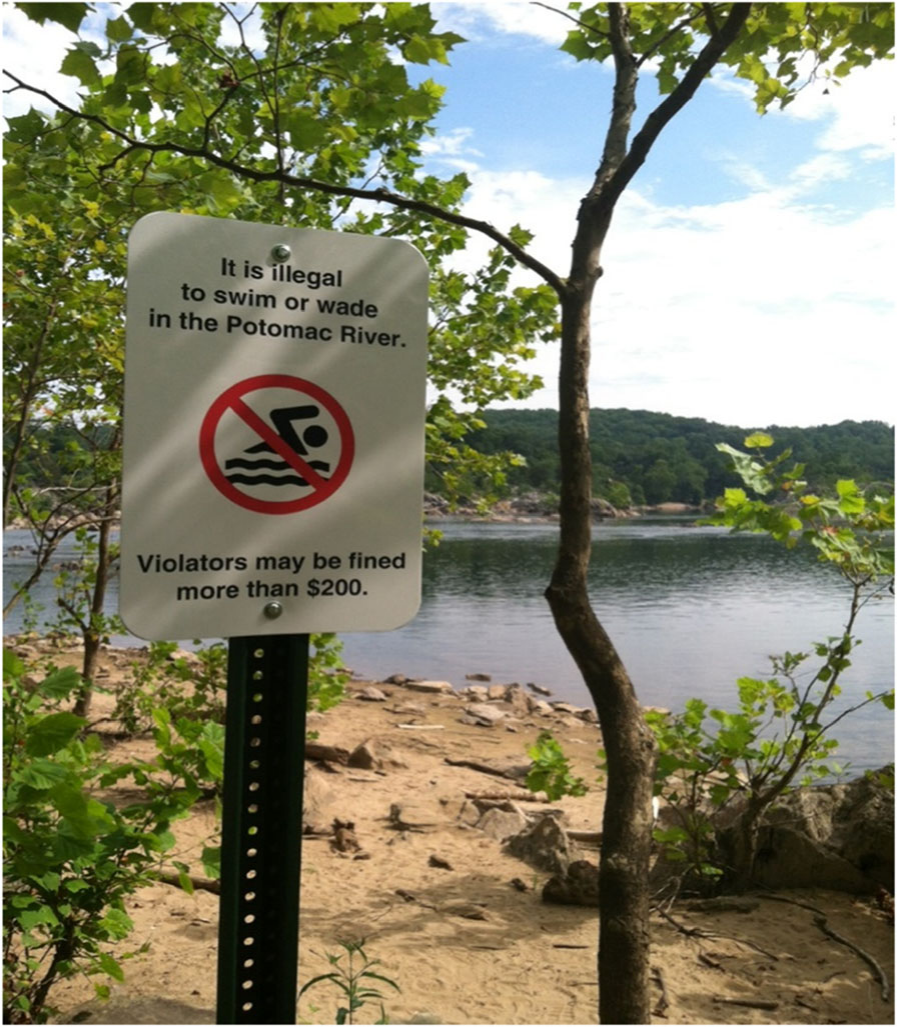
Experimental sign being evaluated

**Fig. 2:**
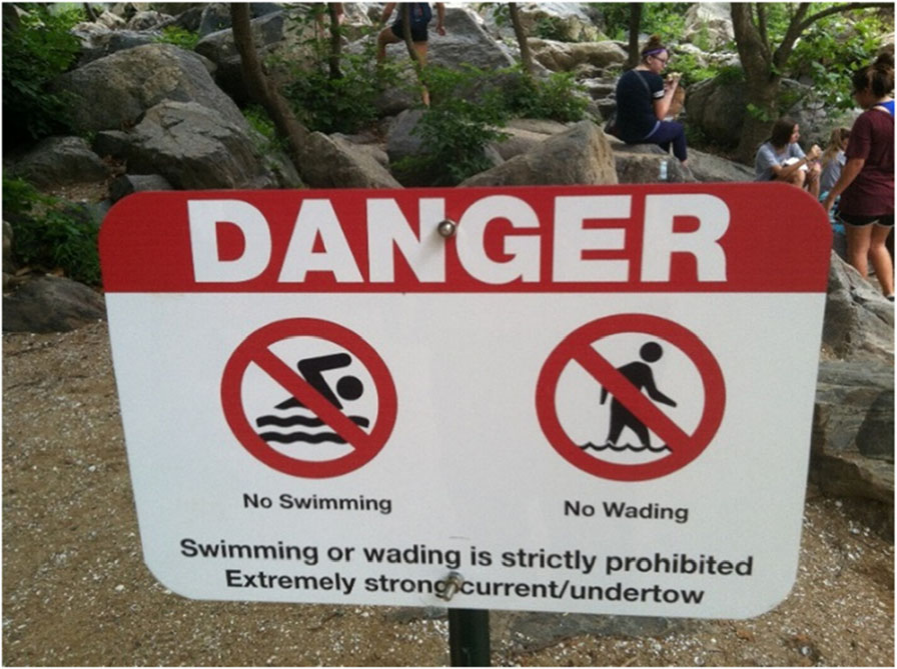
Standard or background sign

In 2015–16, GWMP and CHOH entered into a collaboration with the author, who is a University-based safety researcher with drowning prevention expertise and prior research experience in national parks. Various risk reduction options were discussed, and an agreement was reached to evaluate the effect of a novel sign erected at sites associated with previous risk-taking. The new sign informed park visitors that entering the water was illegal and could result in them incurring a $200+ fine. It was erected in addition to a standard water safety message that emphasized the dangers associated with river entry. So, in this intervention study, park visitors were exposed to either an experimental sign (i.e., see Fig. 1) displayed in addition to a standard sign (i.e., see Fig. 2), or the standard sign alone. Our research plan was reviewed and approved in advance by the National Park Service’s Research Permit and Reporting System, as well as the Institutional Review Board of the Uniformed Services University of the Health Sciences.

### Study procedures

**Fig. 3:**
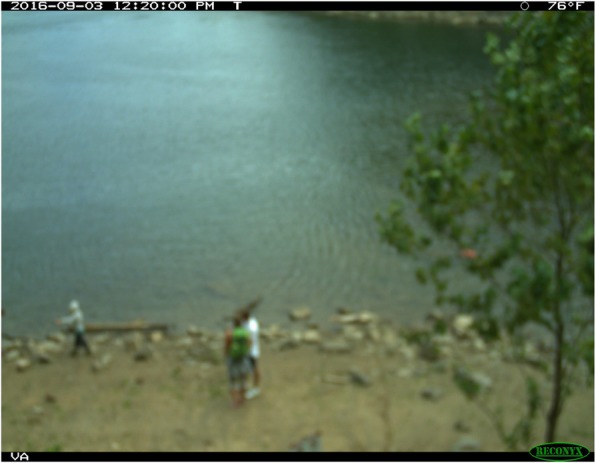
Example of blurred image used to record visitor behavior

Cameras were installed at each study location. We used two Reconyx HyperFire™ Cellular Professional White Flash Camera Traps, which were enclosed in heavy duty security enclosures, secured by ratchet straps and Python locks. Digital images were captured on memory cards, so that behaviors could be observed after they had occurred. The cameras were placed high in trees to discourage vandalism, and capture a view of the “beach” as well as the water. This provided views of visitors who did and did not enter the water. Note that in order to protect any visitors who were photographed entering the water (i.e., an illegal behavior), the focal distance of study cameras were custom-set to one foot (i.e., .3 m). That measure ensured that images would be blurred enough to prevent facial recognition but still allow for subject counts (see Fig. 3).

Two research assistants varied study conditions according to a prescribed schedule and documented environmental conditions (i.e., air temperature, dew point, cloud cover and water level). The latter (i.e. discharge rate) was obtained from the Little Falls Pumping Station Gauge, as posted to the US Geological Survey’s website (http://waterdata.usgs.gov/md/nwis/uv?cb_00060=on&format=gif_stats&site_no=01646500).

Data were collected from July 30, 2016 to September 11, 2016. During this period the experimental sign was displayed on alternating weekend days in each park from approximately 9:30 am to 5:30 pm. Our cameras were programmed to take still photographs, at 10 min intervals throughout the day. Our two data collections sites were Purple Horse Beach (CHOH) and the mouth of Difficult Run stream (GWMP). These sites were selected based upon prior patterns of visitor risk-taking. Small versions (12”× 18”) (.30 m x .46 m) of our aluminum signs were installed along the river’s shore at these locations, and on the trail leading to these locations. PVC versions of the experimental sign, sized 12″ × 9″ (.30 m x .23 m) were installed on the parks’ restroom doors. A large version of the aluminum sign (3’× 6’) (.91 m × 1.83 m), was installed at the park entrances closest to our observation sites.

**Fig. 4:**
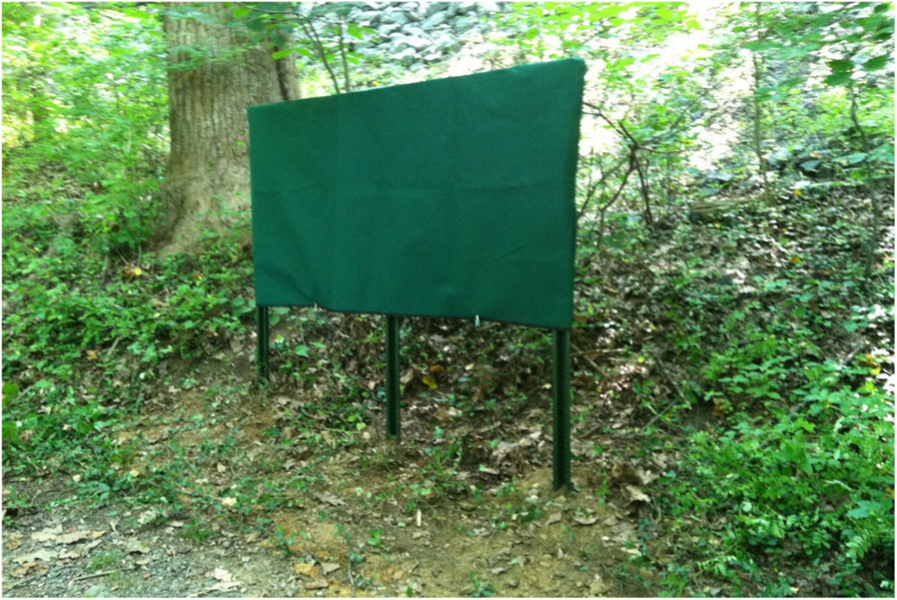
Large version of experimental sign, in covered state

On “control days,” the posted experimental signs were covered (see Fig. 4), leaving the standard (i.e. safety) signs still visible to visitors. Our custom made sign covers were canvas, and had grommets that accommodated padlocks. Our restroom door signs were removed and stored under control conditions. Note that any enforcement actions of the U.S. Park Police were carried out independently of this study or its schedule.

### Data management and analysis

When data collection was ended, our camera equipment was retrieved from the parks, and study images were downloaded onto a password-protected computer. Using software provided by the camera’s manufacturer, they were reviewed in detail on a laptop computer. Data were entered into a SPSS® dataset.

For each captured image (which was our unit of analysis), the reviewer recorded the number of people observed on the beach and the number observed in the water. (Note that people who remained in vessels were not counted, since that behavior is legal on the Potomac River.) For each person observed in the water, a study team member documented degree of water entry (i.e., wading, above their knees but below their waist, above their waist but below their shoulders, or above their shoulders). Visitors who were submerged above their shoulders are counted as swimmers, because we could not determine the depth of the water they were in. Also, given the strength of the Potomac River’s current at our study sites, such people were at considerable risk. If a visitor was observed in more than one image, he or she was counted only once. Their sighting was either attributed to the first time they were seen OR to the image in which they performed their most risky behavior (e.g., water entry). Care was taken to distinguish individuals (e.g., visitor apparel was studied, as were hair styles, color of backpacks, etc).

Temperature was recorded from the reading that was automatically stamped on each image by our camera. Dew point was taken from our field team’s daily notes, which they obtained from a handheld device (i.e., an Oregon Scientific Portable Weather Forecaster). Cloud cover did not demonstrate sufficient variability on study days, therefore it was not included in our analyses.

Once all data were entered, a reliability check was carried out with 10 % of images which were selected using a random numbers table. The author reviewed those photos, without knowledge of what the first reviewer had reported. Reviewers 1 and 2 achieved 100% agreement. Frequencies were then run, and checked for unlikely patterns or missing values. Student’s t tests and Chi Square tests of association were used to identify independent variables that were associated with the presence or absence of people in the water at a *p* < .10 level of significance. Any such variables were then entered into a logistic regression equation to explore their independent associations with the dependent variable. To be retained in our final model, variables had to reach *p* < .05. Finally, a Hosmer and Lemeshow Test was carried out to assess the model’s goodness-of-fit.

## Results

### Descriptive findings

A total of 1441 images were captured and reviewed. Of those, 53% were taken in CHOH and 47% were in GWMP. We found that 1.7% of the images in Maryland and .9% of the images in Virginia showed a visitor in the water. In ten (53%) of the 19 images that showed people in the water, they were there by themselves. Six (32%) of the images showed two people in the water, two photographs (11%) showed three and one (5%) showed four people in the water--which was the highest number of people we observed at one time in the section of the river that our camera captured. A total of 32 people were seen in the water over all study days, in both sites combined. The largest proportion of those visitors (*n* = 13) were considered to be swimming (i.e. submerged above their shoulders), with a similar proportion (*n* = 12) observed to be wading. Table 1 shows how our environmental factors varied, by sign condition.

**Table 1:** Environmental factors: by sign condition

Factor	Experimental *(sanction)* Sign Visible *n* = 770 $\overline{X}$ (SD)	Experimental Sign Covered *n* = 671 $\overline{X}$ (SD)
Mean # of visitors on the beach	1.2 (2.4)	1.3 (2.7)
Air temperature (K)	305.65 (5.25)	305.17 (9.2)
Dewpoint (K)	293.83 (3.49)	293.28 (3.74)*
Water level *(river discharge rate, in cubic meters per second)*	89.11 (61.63)	86.48 (65.97)

*p < .05

### Bivariate findings

The number of people observed on the beach was significantly associated with water entry (t = .59, df = 1439, *p* = .005). Air temperature approached significance (t = 2.15, df = 1439, *p* = .053). The mean temperature registered on the camera when someone was observed to be in the water was 2.3 degrees Kelvin (i.e., 4.2 degrees Fahrenheit) higher than when there were no visitors observed in the river. Sign condition (i.e., experimental + control vs control only) approached significance (x^2^ = 3.70, df = 1, *p* = .055). To quantify that observed difference, .8% of the images taken when our experimental sign was displayed showed someone in the water, while 1.9% of those taken when the sign was covered showed someone in the water. Note that study site, weekend day, time of day, dew point and water level were also tested but found to have no significant association with water entry.

### Multivariate findings

As described above, those variables that met our criterion were entered into a Logistic Regression model to see whether they showed independent association with the outcome: water entry. All three of our variables stayed in the model as indicated in Table 2.

**Table 2:** Variables showing significant association with visitor water entry (*n* = 1441)

Variable	Exponent B (*95% CI*)	*p* value
Air Temp (K)	1.11 (*1.01–1.22*)	.024
Beach count	1.16 (*1.05–1.29*)	.005
Sign Condition *(*i.e.*, novel sign on display)*	.37 (*.14–.99*)	.049

The result of our Hosmer-Lemeshow test was 3.56 (d.f. = 8, *p* = .90), which suggests acceptable fit.

## Discussion

In the past, professionals responsible for managing most recreational areas have emphasized a hazard warning message when trying to promote water safety. This approach is intuitive, but often fails to influence those at highest risk of drowning (i.e., young men). This study suggests that the addition of novel, uncluttered sign which emphasizes possible legal and financial consequences of entering a hazardous river may significantly reduce water entry.

We attempted, by design and placement, to make our experimental sign both conspicuous and salient. Of interest is the fact that it incorporated many of the features that were recommended in a recently published article on “best practice principles for communicating messages in national parks.” (Saunders et al. 2019). This approach is further supported by the fact that compliance was assessed via objective measurement in a real world setting. Due to the relative rarity of fatal drownings, and the limited duration of this study, we could not calculate whether the rate of drownings were reduced. Obviously, however, drowning is preceded by water entry, so these findings are encouraging. This approach is relatively inexpensive and may not necessitate additional staffing (assuming that warning signs are already being posted at sites near water hazards).

Public officials responsible for water safety have been slow to apply evidence-based approaches from the traffic safety arena. Seat belt use in cars, for example, increased from 11 to 86% in the United States, when strong legislation was combined with enforcement and publicity (Nichols and Ledingham 2008; Nichols et al. 2014). Nevertheless, campaigns to increase Personal Flotation Device use among adult boaters for example, rely more on education and persuasive messaging than regulatory approaches. There are many psychological mechanisms--several gender-related--that work against safety-based messaging in recreational settings (Girasek et al. 2016b). Perhaps alternative appeals, such as the one tested in this study, need to be considered by more public health professionals. According to the U.S. Centers for Disease Control and Prevention, a majority of the fatal and non-fatal drownings that involve victims above the age of 14 occur in natural bodies of water (Centers for Disease Control and Prevention 2016).

Future studies could be carried out to elucidate how sanction messages impact different subgroups of the population. Males—the primary risk group for fatal drownings—have been shown to react to risk communication differently than females (Tannenbaum et al. 2015; Espiner and Weiss 2010; Hamilton et al. 2018). Our blurred images, however, did not allow us to establish visitor gender. Larger scale studies could also be carried out to see if rescues and/or fatal drownings are reduced over time when awareness of legal and financial consequences are increased.

The significant association we observed between air temperature and river entry was strikingly similar (i.e., B = 1.06 vs 1.04, when temperature is standardized to Farenheit) to what we observed 3 years earlier in Yosemite National Park (Girasek et al. 2016a). Managers of recreational areas should consider hot days to pose a heightened drowning threat, as has been reported elsewhere (Fralick et al. 2013; Office of the Chief Coroner 2011).

**Fig. 5:**
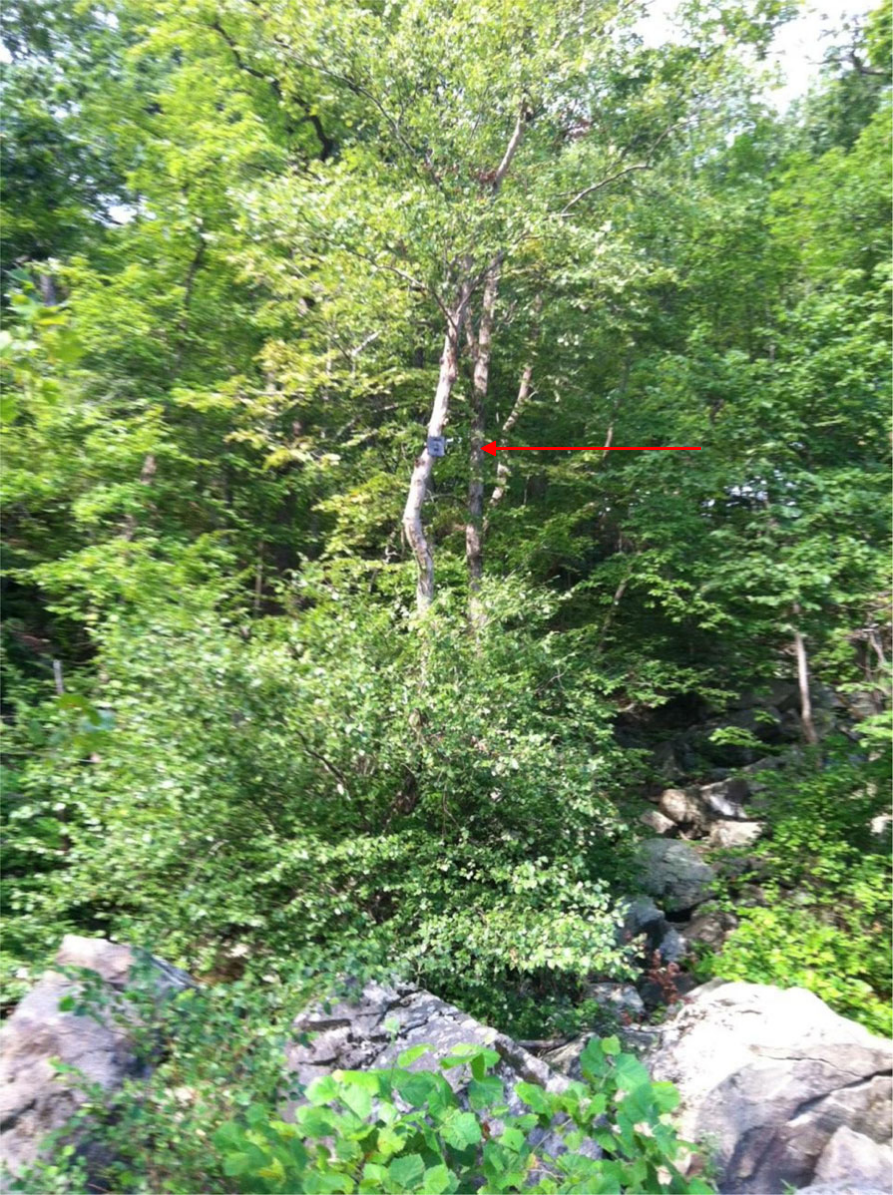
Placement of study camera in tree

This study was subject to limitations. The experimental sign was never tested without a camera present. That might have enhanced its effectiveness, if visitors felt that enforcement was more likely due to surveillance by (perceived) authorities. Any such effect seems unlikely, however, because our cameras were located quite high in their respective trees (see Fig. 5). Since visitors tend to face the river view, their backs would also have been to the camera. We did not have data on law enforcement activities at our study sites on data collection days. Our captured images and field work suggest however, that police patrols did not deviate from standard park operations while our study was in effect.

## Conclusion

It is encouraging that the low cost intervention tested in this study appears to have decreased entries to a dangerous body of water. It is important to note, however that a law which sanctioned water entry had been put in place prior to our study. Existing regulations levied a hefty fine. Safety advocates hoping to apply these findings may face a multi-step process of advocacy aimed at policy makers, followed by public awareness efforts. This work is a reminder that prevention efforts which operate on more than one level—in this case targeting both the person at risk and the regulatory arena--are more likely to be successful than programs which rely on a single component.

## Data Availability

The dataset that was generated by the current study would be made available from the corresponding author upon reasonable request, as long as the IRB of record and funder concur that it may be shared.

## Abbreviations

CFR: Code of Federal Regulations
CHOH: Chesapeake and Ohio National Historical Park
GWMP: George Washington Memorial Parkway
K: Kelvin
NPS: National Park Service
PVC: Polyvinyl chloride
SD: Standard deviation
SPSS: Statistical Package for Social Sciences
